# Dynamic monitoring of membrane nanotubes formation induced by vaccinia virus on a high throughput microfluidic chip

**DOI:** 10.1038/srep44835

**Published:** 2017-03-20

**Authors:** Min Xiao, Na Xu, Cheng Wang, Dai-Wen Pang, Zhi-Ling Zhang

**Affiliations:** 1 Key Laboratory of Analytical Chemistry for Biology and Medicine (Ministry of Education), College of Chemistry and Molecular Sciences, State Key Laboratory of Virology, and Wuhan Institute of Biotechnology, Wuhan University, Wuhan 430072, P. R. China; 2 Institute of Biology and Medicine, Wuhan University of Science and Technology, Wuhan 430065, P. R. China

## Abstract

Membrane nanotubes (MNTs) are physical connections for intercellular communication and induced by various viruses. However, the formation of vaccinia virus (VACV)-induced MNTs has never been studied. In this report, VACV-induced MNTs formation process was monitored on a microfluidic chip equipped with a series of side chambers, which protected MNTs from fluidic shear stress. MNTs were formed between susceptible cells and be facilitated by VACV infection through three patterns. The formed MNTs varied with cell migration and virus concentration. The length of MNTs was positively correlated with the distance of cell migration. With increasing virus titer, the peak value of the ratio of MNT-carried cell appeared earlier. The immunofluorescence assay indicated that the rearrangement of actin fibers induced by VACV infection may lead to the formation of MNTs. This study presents evidence for the formation of MNTs induced by virus and helps us to understand the relationship between pathogens and MNTs.

Membrane nanotubes (MNTs), thin tethers with a diameter of 50~200 nm between neighboring cells or remote cells, have been identified in various types of cells, including neuronal cells, immune cells, and epithelial cells^1^,^2^,^3^,^4^,^5^. MNTs have been estimated as an efficient mode of long-range cell-cell communication, especially for the transfer of electrical calcium-mediated signals^6^,^7^, the transport of intracellular cargos^8^,^9^, and the transmission of bacteria, virus, and prion^10^,^11^. Viruses, such as human immunodeficiency virus (HIV) and murine leukemia virus (MLV), have been demonstrated to induce the MNTs formation by drawing actin-rich filopodial protrusions^12^,^13^. Vaccinia virus (VACV), a member of *Orthopoxvirus* with a large DNA genome, has been reported that it can induce susceptible cells to produce long filopodia or protrusions^14^. So, we wondered if VACV can promote MNTs formation. To test this hypothesis, we studied the VACV-host interaction by monitoring the morphology change of VACV-infected cells in this report.

Nevertheless, Fragile MNTs are very sensitive to mechanical stress and light^2^. These features impel us to create a controllable cell culture microenvironment for the reliable quantitative determination of MNTs. Microfluidics is an effective technique to control physical and chemical environment of mammalian cells due to the comparable size of microchannel to cell, the high-throughput analysis ability^15^,^16^,^17^, and the powerful integration capacity with fluidic, electric, optic, and magnetic fields^18^,^19^,^20^. Virus-host interactions have been investigated on microfluidic chips. A microgrooved substrate to guide cell alignment has been fabricated to mimic the extracellular matrix topography and explore the virus cell-to-cell spread behavior^21^. Single PC12 cells have been patterned orderly on biofunctionalized gold microstructures to analyze the influence of cell spacing and alignment to the formation of MNTs^22^. Optical tweezers on microfluidic chip were used to pull out MNTs^23^. Shear-free microenvironment can be fabricated by designing variable cell culture chambers, such as side chamber or three-dimensional chamber. The infection process of Pseudorabies virus has been monitored *in situ* on a microfluidic chip with three-dimensional cell culture chambers^16^.

Herein, we integrated the high-throughput side cell culture chambers with a tree-like concentration gradient generation network on a microfluidic chip to monitor the formation of MNTs induced by VACV. The side-chambers supply a shear-free culture circumstance for mammalian cells which is beneficial to decrease the mechanical harm to MNTs. Thus, the analysis of VACV-induced MNTs derived from the microfluidic chip is more reliable. We observed three formation patterns of MNTs, analyzed the change of MNTs characteristic with infection time and virus concentration, and further discussed the physical basis of MNTs formation.

## Results and Discussions

### Shear-free culture environment for MNTs

**Figure 1:**
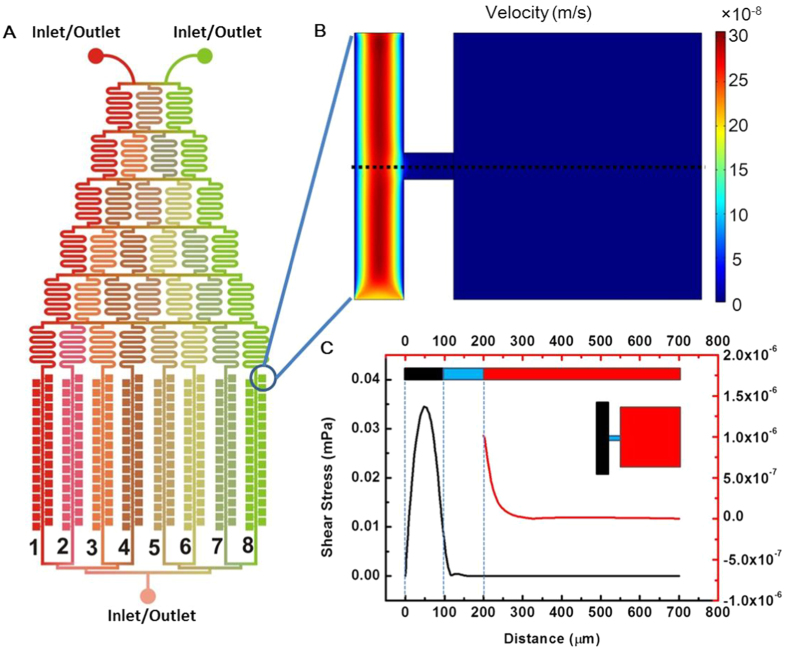
Schematic diagram of the microfluidic chip and the simulation of fluid velocity field in the chip channel. (**A**) The microfluidic chip was composed of a tree-like microchannel network and eight microchannel lists with side-chamber. The chamber (500 μm × 500 μm) was connected with main channel through a narrow channel (50 μm). (**B**) Simulated velocity field in the channel and chamber. (**C**) Shear stress in the middle line of the chamber (dot black line in **B**). The shear stress in the chamber was much lower than that in the main channel.

MNTs are sensitive to mechanical stress, so we designed a microfluidic chip with side-chambers for cell culture (Fig. 1A). By simulating the fluidic velocity distribution with finite element analysis (Fig. 1B), we estimated the fluidic shear stress in the chamber. The shear stress in the middle line of the chamber (dot black line in Fig. 1B) was shown in Fig. 1C. When setting the velocity in the main channel as 20 μL/h, the maximal shear stress in the main channel was about 0.35 mPa. The shear stress in the side chamber was at the range of 9.8 × 10^−7^ mPa~1.7 × 10^−10^ mPa and decreased rapidly with the distance departing from the side exit of the main channel. Compared to other chips^24^,^25^,^26^, the shear stress acting on cells in our chip was negligibly small. Simultaneously, each side-chamber was independent and non-interfering. This created a shear-free culture environment and high-throughput platform for the investigation of MNTs.

### Formation patterns of VACV-induced MNTs

**Figure 2:**
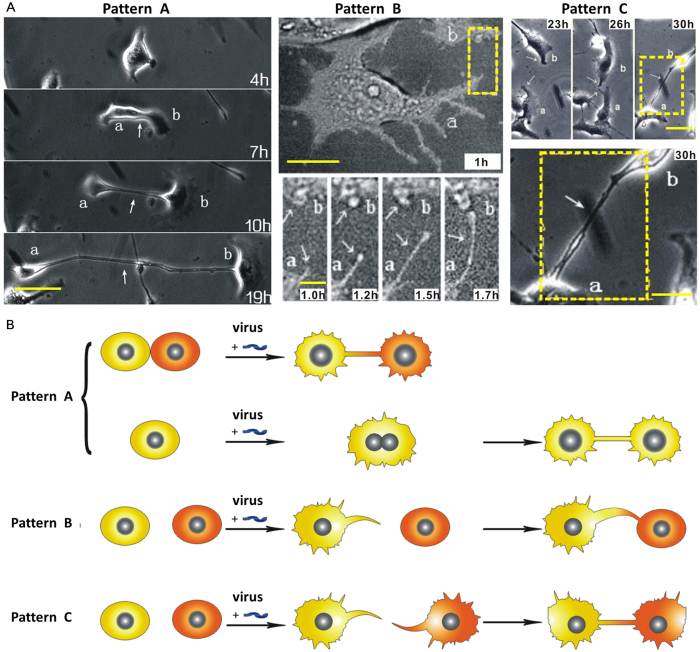
Formation patterns of MNTs. (**A**) Three pairs of cells with MNTs formed through the three distinct patterns. The first group of images showed that MNT formed when two adjacent cells move apart (Pattern A). The second group of images showed the formation of MNT through a protrusion. The third group of images showed that MNT formed when two protrusions from two distinct cells meet randomly and connect. Scale bars, left column, 20 μm; middle column top row, 20 μm, bottom row, 5 μm; right column top row, 30 μm, bottom, 10 μm. (**B**) Schematic diagram of MNTs formation pattern.

To study VACV-induced MNTs, MNTs between normal Vero cells was used as control. Virus suspension was injected into the chip for 20 min to ensure that all cells in the chambers were infected. The formation process of VACV-induced MNTs was monitored and classified into three patterns (Fig. 2). Three representative pairs of infected cells were shown in Fig. 2A. For Pattern A, MNTs can be drawn out when two contacted cells or divisive daughter cells move apart. For Pattern B, a protrusion from one cell extends to a nearby cell for the formation of MNT. For Pattern C, two protrusions derived from distinct cells meet randomly and subsequently connect to form MNT.

**Figure 3:**
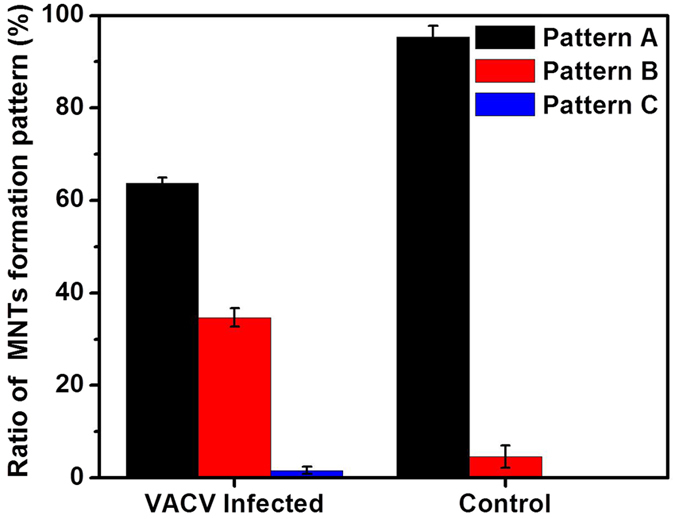
Ratio of each formation pattern of MNTs between infected cells and normal cells (control condition).

The ratios of three formation patterns of MNTs were measured (Fig. 3). The result showed that, both in the conditions of VACV infection and control, the ratios of Pattern A, B, and C decreased in order. The ratio of Pattern A in VACV infection was (63.7 ± 1.2)%, down from (95.4 ± 2.4)% in the control condition. The ratio of Pattern B in VACV infection increased from (4.6 ± 2.4)% in control condition to (34.7 ± 2.0)%. Pattern C was not observed between normal cells in the control condition. Among the three Patterns, B and C can be considered as active formation, while A is passive formation.

### MNTs elongation with cell migration

**Figure 4:**
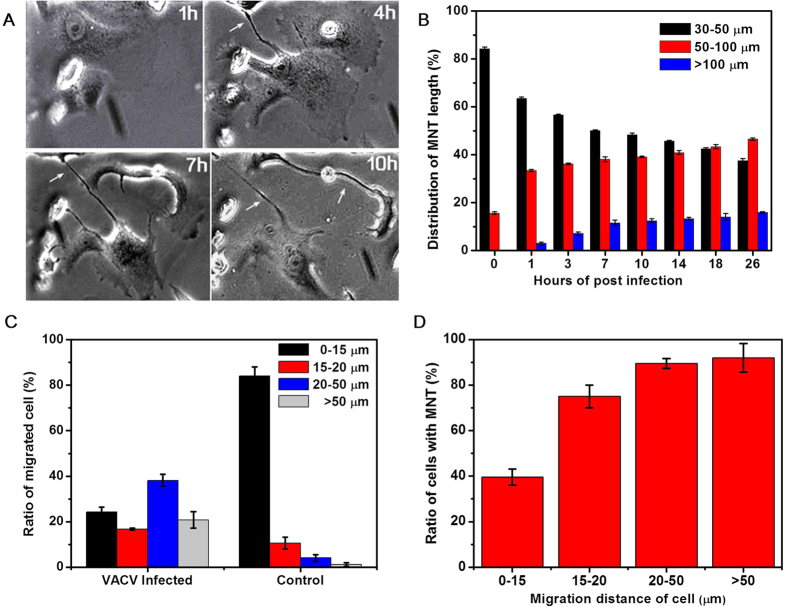
MNTs elongation and cell migration. (**A**) MNTs between infected cells were elongated with infection time. (**B**) Distribution of MNTs length with infection time. MNTs length was divided into three ranges: 30–50 μm, 50–100 μm, and >100 μm. The ratio of long MNTs increased with infection time. (**C**) Ratio of migrated cell when cells were infected by VACV compared with normal cells (control). The three ranges represented the distance of cell migration. (**D**) The relationship of cell migration distance and ratio of cells with MNTs.

The structure of MNTs is dynamic and varies with surrounding circumstances^1^. It was found that some VACV-induced MNTs elongated remarkably with the infection process (Fig. 4A). The length of MNTs at different hours of post infection (hpi) was measured to estimate the elongation (Fig. 4B). The length of MNTs was divided into three ranges: 30–50 μm, 50–100 μm, and >100 μm. Before infection, (84.3 ± 0.6)% MNTs was in the range of 30–50 μm and all MNTs length was less than 100 μm. The ratio of long MNTs increased and the ratio of short MNTs decreased with infection time. At 26 hpi, the ratio of MNT length in the range of 30–50 μm was reduced to (37.5 ± 1.0)%, and the ratio of >100 μm MNT reached to (15.9 ± 0.4)%.

In the above discussion of MNTs formation pattern, the formation of MNTs is closely related with the behavior of cell migration. VACV infection can result in cell motility, which is driven by the polymerization and depolymerization of actin within filopodia and lamellipodia^14^,^27^. The migration distance of infected cell was divided into four ranges: 0–15 μm, 15–20 μm, 20–50 μm, and >50 μm (Fig. 4C). For the control experiment, most of the cells, about (84.1 ± 3.9)%, migrated within the range of 0–15 μm. For the VACV infection experiment, the ratios of migration distance in the four ranges were (24.3 ± 2.1)%, (16.7 ± 0.5)%, (38.2 ± 2.7)%, (20.8 ± 3.6)%, respectively. The result showed that infected cells migrated longer than normal cells. The proportion of migrated cells with MNTs (Fig. 4D) showed that the ratio of cells with MNTs was positively correlated with the distance of cell migration. These results suggested that the elongation of VACV-induced MNTs was caused by cell migration.

**Figure 5:**
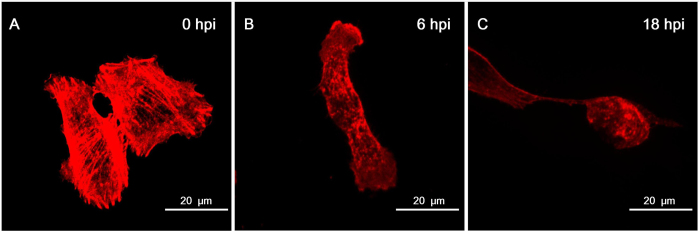
Rearrangement of actin network in Vero cells with VACV infection. F-actin component was labeled with Alexa Fluor^®^ 568 phalloidin (red). (**A**) Long and thick actin fibers were across the whole cell in normal Vero cells. (**B**) Actin fibers disappeared and short actin filaments appeared at 6 hpi in VACV-infected cells. (**C**) Actin filaments were still dispersive and the shape of infected cells was changed into round at 18 hpi. (MOI = 10).

Some studies proved that the growth and stability of MNTs is closely related with actin filaments^28^,^29^. Growing actin filaments push the MNTs outward and search for the surrounding environment. Previous studies showed that VACV infection induces the rearrangement of actin network^30^,^31^. We labeled F-actin with fluorescent phalloidine and found that VACV-induced MNTs were rich of F-actin. The depolymerization of actin fibers in Vero cells was observed early in infection (Fig. 5) as previously presented in PtK2 cells^30^. As a component of cytoskeleton to support cellular mechanical property, the disappearance of actin fibers across the cell made the plasma membrane more flexible. Thus, more protrusions would appear in VACV-infected cells and further actively search for target cells for the formation of MNTs.

### Effect of virus concentration on MNTs

**Figure 6:**
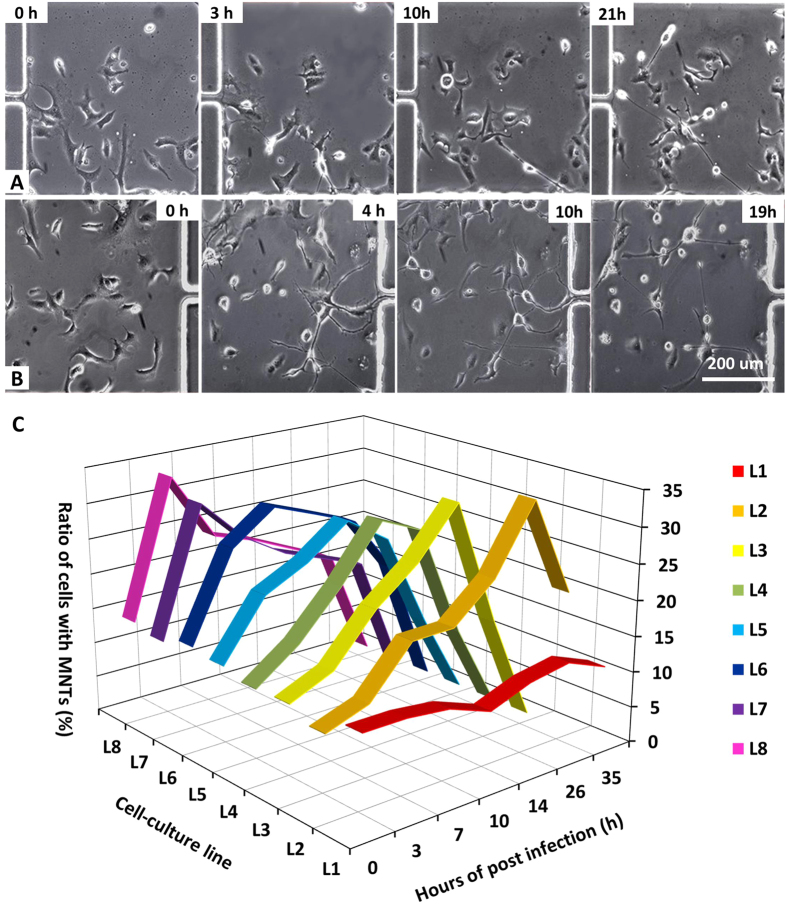
Influence of virus concentrations on MNTs formation. (**A**) and (**B**) were images of cell morphology with infection time at L2 (virus titer was 1.4 × 10^6^ PFU/mL) and L8 (virus titer was 1.0 × 10^6^ PFU/mL), respectively. (**C**) Ratio of cells with MNTs varies with virus titer and infection time. The titers of virus at L2, L3, L4, L5, L6, L7 and L8 were 1.4 ×1 0^5^ PFU/mL, 2.9 × 10^5^ PFU/mL, 4.3 × 10^5^ PFU/mL, 5.7 × 10^5^ PFU/mL, 6.9 × 10^5^ PFU/mL, 8.6 × 10^5^ PFU/mL, 1.0 × 10^6^ PFU/mL, respectively. As a control condition, there was no virus at L1.

To evaluate the influence of virus concentration on the formation of MNT, we used the microfluidic chip to generate different virus concentrations. Figure 6A and B showed the changes of cell morphology in the chambers of List 2 with the virus titer of 1.4 × 10^5^ PFU/mL and in the chambers of List 8 with the virus titer of 1.0 × 10^6^ PFU/mL. With the increase of virus concentration, more protrusions and MNTs were drawn out. In List 2, cells changed their morphology gently with infection time and long MNTs began to form at about 10 hpi. In List 8, cells began to produce protrusions as early as 1 hpi and MNTs were observed at 4 hpi.

The ratios of cells with MNTs after VACV infection at different titers were measured (Fig. 6C). Cells in List 1 were negative control as there is no virus in this list. The ratio of cells with MNTs was in the range of 10%~15% in the control condition, which is less than the infected cells. For infected cells, the ratio of cells with MNTs initially increased with infection time, then reached a peak, and decreased soon afterwards. The peak values at different virus concentration were almost the same, about 30%. But the time to reach the peak varied with virus concentration. When the virus concentration increased, the time to reach the peak gradually became early (Table 1). This is because one cell can be infected by multiple virus particles when virus titer is high enough and host cells respond to virus invasion rapidly. These results suggested that the phenotype of VACV-induced MNTs was significantly influenced by virus titer.

## Conclusions

Here, we reported the formation of MNTs induced by VACV on a high-throughput microfluidic chip. The side-chamber on the chip supplied a shear-free microenvironment for the host cell to avoid the mechanical stress. MNTs were observed to form between infected cells trough three patterns: two contacted cells move apart (Pattern A), or protrusions from one cell extend towards another remote cell (Pattern B), or two protrusions from distinct cells occasionally encounter and connect (Pattern C). Compared with normal cells, the ratio of Pattern B and C between infected cells increased and the ratio of Pattern A decreased. Simultaneously, MNTs were elongated with the motility of infected cells and the infection time. The length of MNTs was positively correlated with cellular migration distance. We discussed the physical basis of MNTs formation. Cellular actin filaments were responsible for the formation and stability of MNTs. Furthermore, the characteristic of MNTs was influenced by the concentration of VACV. When the virus titer was enhanced, the appearance of MNTs and the highest ratio of cells with MNTs became earlier. The highest ratio of MNT-carried cells was approximately the same at different virus concentrations. Overall, this work presents evidence in favor of VACV-induced formation of MNTs, which provide further insight into the mechanism of virus transmission.

## Materials and Methods

### Chip design and fabrication

A tree-like microchannel network and an array of side-chamber for cell culture was combined on a microfluidic chip (Fig. 1A). There were 320 square cell culture chambers (length × width: 500 μm × 500 μm) on the chip for high throughput analysis, with 40 chambers on each channel.

The microfluidic chip was fabricated by the standard soft lithography method. First, the AZ50XT photoresist was spin-coated on a silicon wafer at a speed of 1200 rpm. After exposure with a mask under UV light, the photoresist was developed in an AZ developer to obtain the designed mould. Polydimethylsiloxane (PDMS) prepolymer and curing agents were mixed at a ratio of 10:1 (w/w). And then the degassed PDMS was poured onto the mould for further curing at 75 °C. The solid PDMS was peeled off and irreversibly sealed with a glass slide after plasma treatment.

### Simulation of fluid velocity filed in the microfluidic chip

The fluid velocity field was simulated with Comsol Multiphysics software (version 4.0). The basic assumption of the theoretical model and the evaluation of shear stress were presented as follows.

The chip was simplified to be two-dimensional and the fluid was assumed to be laminar and incompressible Newtonian fluid. Accordingly, the Navier–Stokes equations, eqs (1) and (2), were used to solve the velocity distribution. A no-slip boundary condition at the inner wall of the chamber and zero outlet pressure were taken into consideration.


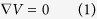



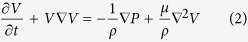


where V is the velocity vector (m · s^−1^), P is the pressure (Pa), and *μ*/*ρ* is the kinematic viscosity (m^2^ · s^−1^). The working fluid was assumed to be water (a homogeneous, incompressible, Newtonian fluid; density 1.0 × 10^3^ kg · m^−3^, dynamic viscosity 1.0 × 10^−3^ Pa · s).

For 2D Poiseuille flow, the shear stress at the wall (τ_wall_, dyn·cm^−2^) can be estimated simply by equation (3), which derives from the Navier–Stokes equations.


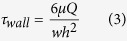


where Q is the flow rate (m^3^·s^−1^), μ is the fluid viscosity (kg·m^−1^·s^−1^), and *w* and *h* are the channel width and height (m), respectively.

### Cell culture and virus infection

African green monkey kidney cells (Vero cells) were cultured in Dulbecco’s Modified Eagle’s Medium (DMEM, Gibco) with 10% fetal bovine serum (FBS, Gibco)(named as culture medium). Vero cell suspension (5.0 × 10^5^ cell/mL) was injected into the microfluidic chip and 20~40 cells were captured in each chamber. Then, the microfluidic chip was settled in an incubator with 5% CO_2_ overnight to cultivate the cells. A recombinant vaccinia virus (VACV) expressing green fluorescent protein (GFP) was kindly supplied by Wuhan Institute of Virology (Chinese Academy of Sciences, Wuhan, China). All experiments about virus were performed in DMEM with 2% FBS (named as maintenance medium).

To explore the formation pattern and length distribution of VACV-induced MNTs, virus suspension (1.0 × 10^6^ PFU/mL) was injected from the single inlet near the cell culture region at a flow rate of 0.5 μL/min and maintained for 20 min. Injected virus suspension was incubated at 37 °C for 2 hours. After the virus incubation, fresh maintenance medium was perfused into the chip. Cells without virus infection were used as the control experiment.

To explore the effect of different virus concentrations on the formation of MNTs, the virus suspension and maintenance medium were perfused into the microfluidic device from the two inlets near the microchannel region, respectively. According previous studies^32^, setting the highest virus concentration in List 8 as C, the theoretical virus concentrations from list 1 (L1) to list 8 (L8) were 0, 1/7C, 2/7C, 3/7C, 4/7C, 5/7C, 6/7C and C, respectively.

### Dye staining and immunofluorescence labeling

To visualize actin cytoskeleton, immunofluorescence experiments were performed as follows. First, cells were washed with phosphate buffered saline (PBS) for 3 times and fixed with 4% (w/v) paraformaldehyde for 15 min. Then, the cells were exposed in PBS with 0.01% Triton X-100 and 5% BSA for 30 min. Subsequently, Alexa Fluor^®^ 568 phalloidin (EX/EM = 578 nm/600 nm, Invitrogen, USA) was added to label F-actin at room temperature for 20 min. Then, cellular nucleus was stained with 5 μg/mL Hoechst 33342 (Invitrogen) at room temperature for 10 min. To observe the rearrangement of actin cytoskeleton, vaccinia virus (VACV) was added to cells at the MOI of 10. F-actin was stained as the above description at 0 hpi (hours of post infection), 6 hpi, and 18 hpi.

### Cell imaging and statistical analysis

**Figure 7:**
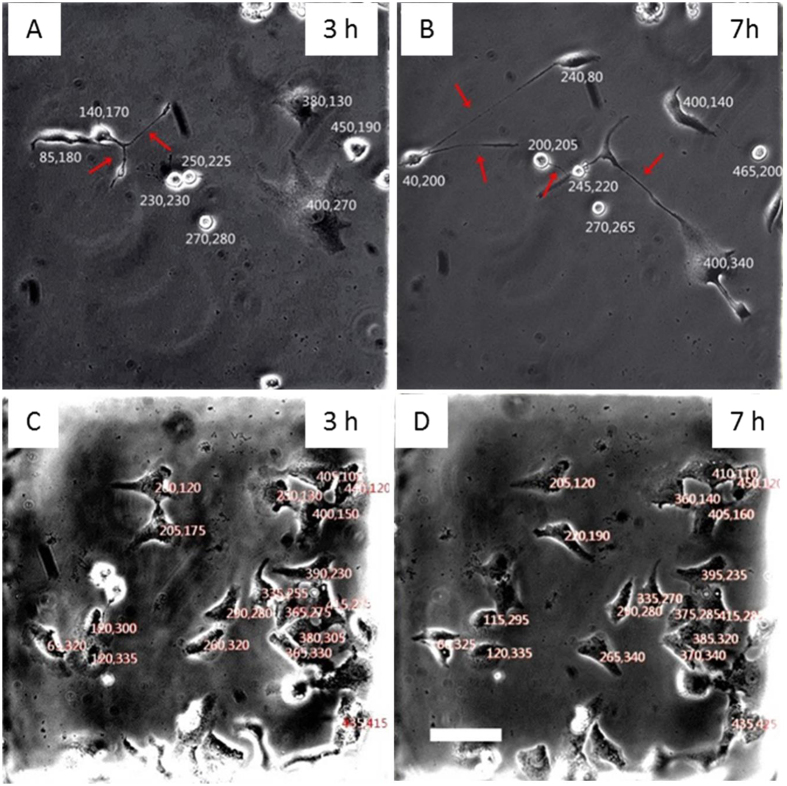
Relative coordinates for labeling cell location. (**A**) and (**B**) showed cells infected by VACV for 3 hours and 7 hours, respectively. (**C**) and (**D**) showed normal cells at 3 hour and 7 hour after the observation begins. Each cell was given a relative coordinate, so cell migration distance was calculated by measuring the variation of coordinates. Scale bar: 100 μm.

Cells were imaged at certain time intervals after VACV infection by an inverted microscopy (TE2000-U, Nikon, Japan) with a CCD camera (Retiga 2000R, Qimaging, Canada). Image Pro-plus software (Version 6.2) was used to measure the length of MNTs. To acquire behaviors of cell migration with/without VACV infection, the images of cell culture chambers were converted to bitmap. Each cell was given a relative coordinate corresponding to the top left corner (0, 0). To minimize the errors resulting from cell spread, we chose the center of cell nuclear as labeling location coordinate as shown in Fig. 7. The distance of cell migration was calculated by analyzing the variation of cell coordinate. All results were from three parallel experiments.

## Additional Information

**How to cite this article**: Xiao, M. *et al*. Dynamic monitoring of membrane nanotubes formation induced by vaccinia virus on a high throughput microfluidic chip. *Sci. Rep.* **7**, 44835; doi: 10.1038/srep44835 (2017).

**Publisher's note:** Springer Nature remains neutral with regard to jurisdictional claims in published maps and institutional affiliations.

## Figures and Tables

**Table 1:** MNTs variation with virus concentration.

List No	Virus concentration (PFU/mL)	Highest ratio of cell with MNTs (%)	Time to reach the highest ratio of cell with MNTs (hpi)
L1	0	13.34 ± 0.56	—
L2	1.4 × 10^5^	33.66 ± 1.50	26
L3	2.9 × 10^5^	33.06 ± 1.85	14
L4	4.3 × 10^5^	30.33 ± 2.01	10
L5	5.7 × 10^5^	28.79 ± 1.96	10
L6	7.1 × 10^5^	30.44 ± 0.99	7
L7	8.6 × 10^5^	30.63 ± 1.63	3
L8	1.0 × 10^6^	32.89 ± 2.34	3

## References

[b1] Davis D. M. & Sowinski S. Membrane nanotubes: dynamic long-distance connections between animal cells. Nat Rev Mol Cell Bio 9, 431–436 (2008). 18431401 10.1038/nrm2399

[b2] Rustom A., Saffrich R., Markovic I., Walther P. & Gerdes H. H. Nanotubular highways for intercellular organelle transport. Science 303, 1007–1010 (2004). 14963329 10.1126/science.1093133

[b3] Seyed-Razavi Y., Hickey M. J., Kuffova L., McMenamin P. G. & Chinnery H. R. Membrane nanotubes in myeloid cells in the adult mouse cornea represent a novel mode of immune cell interaction. Immunol Cell Biol 91, 89–95 (2013). 23146944 10.1038/icb.2012.52

[b4] Onfelt B., Nedvetzki S., Yanagi K. & Davis D. M. Cutting edge: membrane nanotubes connect immune cells. J Immunol 173, 1511–1513 (2004). 15265877 10.4049/jimmunol.173.3.1511

[b5] Wang Z. G. . Myosin-driven intercellular transportation of wheat germ agglutinin mediated by membrane nanotubes between human lung cancer cells. ACS Nano 6, 10033–10041 (2012). 23102457 10.1021/nn303729r

[b6] Smith I. F., Shuai J. W. & Parker I. Active generation and propagation of Ca^2+^ signals within tunneling membrane nanotubes. Boiphys J 100, 37–39 (2011). 10.1016/j.bpj.2011.03.007 PMC3077701 21504718

[b7] Wang X. & Gerdes H. H. Long-distance electrical coupling via tunneling nanotubes. BBA-Biomembranes 1818, 2082–2086 (2012). 21930113 10.1016/j.bbamem.2011.09.002

[b8] He K. . Intercellular transportation of quantum dots mediated by membrane nanotubes. ACS Nano 4, 3015–3022 (2010). 20524630 10.1021/nn1002198

[b9] Onfelt B. . Structurally distinct membrane nanotubes between human macrophages support long-distance vesicular traffic or surfing of bacteria. J Immunol 177, 8476–8483 (2006). 17142745 10.4049/jimmunol.177.12.8476

[b10] Gousset K. . Prions hijack tunnelling nanotubes for intercellular spread. Nat Cell Biol 11, 328–336 (2009). 19198598 10.1038/ncb1841

[b11] Sherer N. M. & Mothes W. Cytonemes and tunneling nanotubules in cell-cell communication and viral pathogenesis. Trends Cell Biol 18, 414–420 (2008). 18703335 10.1016/j.tcb.2008.07.003 PMC2628975

[b12] Lehmann M. J., Sherer N. M., Marks C. B., Pypaert M. & Mothes W. Actin- and myosin-driven movement of viruses along filopodia precedes their entry into cells. J Cell Biol 170, 317–325 (2005). 16027225 10.1083/jcb.200503059 PMC2171413

[b13] Sowinski S. . Membrane nanotubes physically connect T cells over long distances presenting a novel route for HIV-1 transmission. Nat Cell Biol 10, 211–219 (2008). 18193035 10.1038/ncb1682

[b14] Morales I. . The vaccinia virus F11L gene product facilitates cell detachment and promotes migration. Traffic 9, 1283–1298 (2008). 18485055 10.1111/j.1600-0854.2008.00762.x

[b15] Ruan J. . Fabrication of a microfluidic chip containing dam, weirs and gradient generator for studying cellular response to chemical modulation. Mat Sci Eng C 29, 674–679 (2009).

[b16] Xu N. . A microfluidic platform for real-time and *in situ* monitoring of virus infection process. Biomicrofluidics 6, 034122 (2012). 10.1063/1.4756793 PMC3470601 24073185

[b17] Ye N. N., Qin J. H., Shi W. W., Liu X. & Lin B. C. Cell-based high content screening using an integrated microfluidic device. Lab Chip 7, 1696–1704 (2007). 18030389 10.1039/b711513j

[b18] Kovarik M. L. . Micro total analysis systems for cell biology and biochemical assays. Anal Chem 84, 516–540 (2012). 21967743 10.1021/ac202611x PMC3264799

[b19] Whitesides G. M., Ostuni E., Takayama S., Jiang X. & Ingber D. E. Soft lithography in biology and biochemistry. Annu Rev Biomed Eng 3, 335–373 (2001). 11447067 10.1146/annurev.bioeng.3.1.335

[b20] Salieb-Beugelaar G. B., Simone G., Arora A., Philippi A. & Manz A. Latest Developments in Microfluidic Cell Biology and Analysis Systems. Anal Chem 82, 4848–4864 (2010). 20462184 10.1021/ac1009707

[b21] Xu N. . Anisotropic cell-to-cell spread of vaccinia virus on microgrooved substrate. Biomaterials 35, 5049–5055 (2014). 24685266 10.1016/j.biomaterials.2014.03.019

[b22] Abel M. P. . Microstructured platforms to study nanotube-mediated long-distance cell-to-cell connections. Biointerphases 6, 22–31 (2011). 21428692 10.1116/1.3567416

[b23] Pascoal P., Kosanic D., Gjoni M. & Vogel H. Membrane nanotubes drawn by optical tweezers transmit electrical signals between mammalian cells over long distances. Lab Chip 10, 2235–2241 (2010). 20661503 10.1039/c004659k

[b24] Vartanian K. B., Kirkpatrick S. J., Hanson S. R. & Hinds M. T. Endothelial cell cytoskeletal alignment independent of fluid shear stress on micropatterned surfaces. Biochem Bioph Res Co 371, 787–792 (2008). 10.1016/j.bbrc.2008.04.167 18471992

[b25] Wang L. . Patterning cells and shear flow conditions: Convenient observation of endothelial cell remoulding, enhanced production of angiogenesis factors and drug response. Lab Chip 11, 4235–4240 (2011). 22051695 10.1039/c1lc20722a

[b26] Bianchi E., Molteni R., Pardi R. & Dubini G. Microfluidics for *in vitro* biomimetic shear stress-dependent leukocyte adhesion assays. J Biomech 46, 276–283 (2013). 23200903 10.1016/j.jbiomech.2012.10.024

[b27] Sanderson C. M., Way M. & Smith G. L. Virus-induced cell motility. J Virol 72, 1235–1243 (1998). 9445023 10.1128/jvi.72.2.1235-1243.1998 PMC124601

[b28] Veranic P. . Different types of cell-to-cell connections mediated by nanotubular structures. Biophys J 95, 4416–4425 (2008). 18658210 10.1529/biophysj.108.131375 PMC2567924

[b29] Iglic A. . Possible role of flexible red blood cell membrane nanodomains in the growth and stability of membrane nanotubes. Blood Cells Mol Dis 39, 14–23 (2007). 17475520 10.1016/j.bcmd.2007.02.013

[b30] Schepis A., Schramm B., de Haan C. A. & Locker J. K. Vaccinia virus-induced microtubule-dependent cellular rearrangements. Traffic 7, 308–323 (2006). 16497225 10.1111/j.1600-0854.2005.00381.x

[b31] Taylor M. P., Koyuncu O. O. & Enquist L. W. Subversion of the actin cytoskeleton during viral infection. Nat Rev Microbiol 9, 427–439 (2011). 21522191 10.1038/nrmicro2574 PMC3229036

[b32] Jeon N. L., Dertinger S. K. W., Chiu D. T., Choi I. S., Stroock A. D. & Whitesides G. M. Generation of solution and surface gradients using microfluidic systems. Langmuir 16, 8311–8316 (2000).

